# A Live Bio-Therapeutic for Mastitis, Containing *Lactococcus lactis* DPC3147 With Comparable Efficacy to Antibiotic Treatment

**DOI:** 10.3389/fmicb.2019.02220

**Published:** 2019-09-27

**Authors:** Michael Kitching, Harsh Mathur, James Flynn, Noel Byrne, Pat Dillon, Riona Sayers, Mary C. Rea, Colin Hill, R. Paul Ross

**Affiliations:** ^1^Teagasc Food Research Centre, Moorepark, Cork, Ireland; ^2^APC Microbiome Ireland, University College Cork, Cork, Ireland; ^3^Teagasc Animal and Grassland Research and Innovation Centre, Moorepark, Cork, Ireland; ^4^School of Microbiology, University College Cork, Cork, Ireland

**Keywords:** mastitis, emulsion, lacticin 3147, somatic cell counts, antibiotics

## Abstract

Bovine mastitis is an ongoing significant concern in the dairy and agricultural industry resulting in substantial losses in milk production and revenue. Among the predominant etiological agents of bovine mastitis are *Staphylococcus aureus*, *Streptococcus uberis, Streptococcus dysgalactiae*, and *Escherichia coli.* Currently, the treatment of choice for bovine mastitis involves the use of commercial therapeutic antibiotic formulations such as Terrexine^TM^, containing both kanamycin and cephalexin. Such antibiotics are regularly administered in more than one dose resulting in the withholding of milk for processing for a number of days. Here, we describe the optimization of a formulation of *Lactococcus lactis* DPC3147, that produces the two-component bacteriocin lacticin 3147, in a liquid paraffin-based emulsion (formulation hereafter designated ‘live bio-therapeutic’) for the first time and compare it to the commercial antibiotic formulation Terrexine^TM^, with a view to treating cows with clinical/sub-clinical mastitis. Critically, in a field trial described here, this ‘ready-to-use’ emulsion containing live *L. lactis* DPC3147 cells exhibited comparable efficacy to Terrexine^TM^ when used to treat mastitic cows. Furthermore, we found that the *L. lactis* cells within this novel emulsion-based formulation remained viable for up to 5 weeks, when stored at 4, 22, or 37°C. The relative ease and cost-effective nature of producing this ‘live bio-therapeutic’ formulation, in addition to its enhanced shelf life compared to previous aqueous-based formulations, indicate that this product could be a viable alternative therapeutic option for bovine mastitis. Moreover, the single-dose administration of this ‘live bio-therapeutic’ formulation is a further advantage, as it can expedite the return of the milk to the milk pool, in comparison to some commercial antibiotics. Overall, in this field trial, we show that the live bio-therapeutic formulation displayed a 47% cure rate compared to a 50% cure rate for a commercial antibiotic control, with respect to curing cows with clinical/sub-clinical mastitis. The study suggests that a larger field trial to further demonstrate efficacy is warranted.

## Introduction

Mastitis is a persistent and financially important disease in dairy cows chiefly due to the costs associated with antibiotic treatment, discarded milk, reduced milk production and veterinary costs. Furthermore, in the event of antibiotic treatment failure, the cows may have to be culled. One study estimated that mastitis resulted in a net farm profit decrease ranging between $19,132 to $91,552 per herd, predominantly due to culling cows and decreases in milk production (Guimarães et al., 2017). Thus, decreasing the rates of mastitis and finding effective treatment options is crucial for ensuring a healthier herd, minimizing the likelihood of cows with high SCC and overall maximizing the opportunities for making profits. Antibiotic resistance (AMR) is a global problem resulting in higher healthcare-associated costs, treatment failure and deaths (European Centre for Disease Prevention and Control, 2014). Indeed, the WHO (World Health Organization) has cited the overuse of antimicrobials in food-producing animals as a major issue, with respect to transferable antimicrobial resistance (Veterinary Ireland, 2014). It is therefore essential that preventative strategies and alternative treatment plans are devised to reduce reliance on antibiotics in the dairy herd, which is in line with Action 6 of the European Commission’s Roadmap against AMR (European Commission [EC], 2015).

The primary etiological agents of bovine mastitis are *Staphylococcus aureus*, *Streptococcus uberis* and *Streptococcus dysgalactiae* (Crispie et al., 2008; Zadoks et al., 2011; Abebe et al., 2016; Bi et al., 2016). While antibiotics have proved to be effective in some cases against mastitis, antibiotic resistance and agents such as *S. aureus* which may be recalcitrant to antibiotic therapy are major causes for concern (Barkema et al., 2006; Melchior et al., 2006). This frequently results in a pattern of prolonged recurrent infections within a dairy herd (Crispie et al., 2008). To date, other forms of therapies (or combination of antimicrobials) for mastitis have reported varying levels of efficacy. Examples of such therapies include the combination of lactoferrin with the antibiotic penicillin G (Diarra et al., 2002, 2003); bacteriocins such as nisin which resulted in significantly increased cure rates amongst *S. aureus* infected cows (Cao et al., 2007); immune-stimulants such as ginseng (Hu et al., 2001) and also the use of cytokines (Barkema et al., 2006). Viable *Lactobacillus casei* cells to prevent the invasion of *S. aureus* into bovine mammary epithelial cells have also been utilized (Bouchard et al., 2013). More recently, a *Lactococcus* culture, V7, was found to inhibit *Escherichia coli* and *S. aureus* invasion of bovine mammary epithelial cells (Assis et al., 2015). However, further studies have yet to elicit their efficacy *in vivo*. Finally, bacteriophage therapy with a view to treating bovine mastitis has been hindered as a result of the inhibition of phages in bovine milk (O’Flaherty et al., 2005).

We have already shown that the two-component bacteriocin, lacticin 3147, produced by the lactic acid bacterium (LAB) *Lactococcus lactis* DPC3147 inhibits Gram positive mastitis pathogens (Ryan et al., 1998, 1999; Klostermann et al., 2008, 2010). *L. lactis* strains are routinely used as dairy starter organisms and several strains have been granted GRAS (generally regarded as safe) status in the dairy industry. In studies by Ryan et al., and Twomey et al., it was demonstrated that a combination of lacticin 3147 and bismuth-based teat seal prevented *S. dysgalactiae* infection in dry cows (Ryan et al., 1999) and *S. aureus* infection in lactating cows (Twomey et al., 2000). Moreover, a freeze-dried preparation of the lacticin 3147 producing culture resuspended in sterile water was found to be as effective as an antibiotic in curing clinical mastitis (Klostermann et al., 2008). Since a Gram negative, lacticin 3147-insensitive *E. coli* strain was amongst the eliminated pathogens described in a the study by Klostermann and co-workers, it was postulated that some other mechanism besides the bacteriocin was also in play (Klostermann et al., 2008). In addition, it was demonstrated that infusion with freeze-dried *L. lactis* DPC3147 rapidly stimulated the host intra-mammary immune system triggering the influx of lymphocytes and polymorphonuclear leukocytes (PMNs), to the mammary gland along with the localized production of acute phase proteins (APP). These factors in combination help clear the mammary gland of the infecting pathogen (Crispie et al., 2008). In this regard, it was clear that DPC3147 offers substantial potential as a live bio-therapeutic for mastitis treatment, and the production of low concentrations of lacticin 3147 *in vivo* may contribute to the strain’s ability to induce an immune response in the host.

The cost-prohibitive nature of producing sufficient quantities of pure antimicrobial peptides has proved to be a stumbling block for the use of purified antimicrobial bacteriocins to treat bovine mastitis. Therefore, in this study, we decided to further investigate the lacticin 3147-producing organism as a live bio-therapeutic to treat mastitis and to compare it to an antibiotic treatment. We describe the development of a novel liquid paraffin-based emulsion of *L. lactis* DPC3147 (henceforth termed ‘live bio-therapeutic’) with the potential as an alternative treatment option for mastitis. We demonstrate that this ‘live bio-therapeutic’ formulation possesses comparable efficacy to a commercial antibiotic with respect to treating cows exhibiting signs of clinical mastitis and/or cows with sub-clinical mastitis with elevated SCC, abnormal milk samples and presence of pathogens in milk.

## Materials and Methods

### Preparation of *Lactococcus lactis* DPC3147 Culture

**FIGURE 1:**
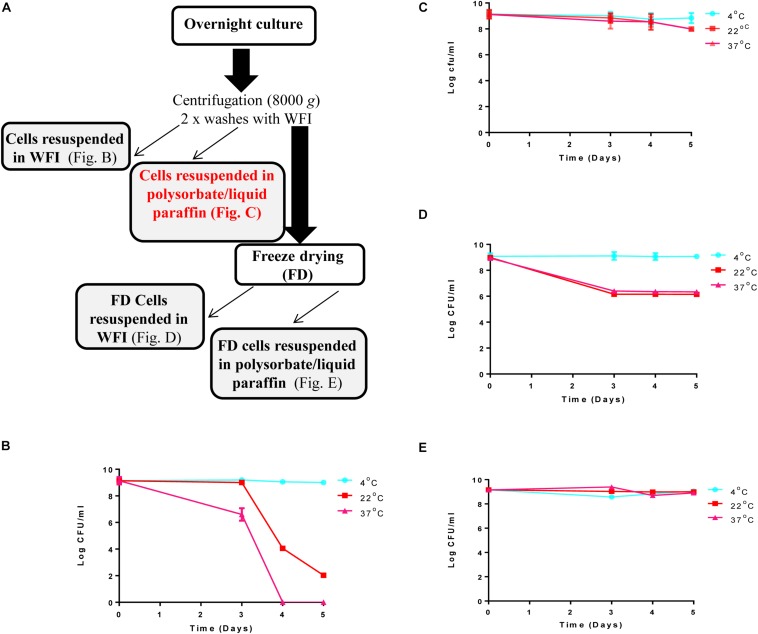
Steps involved in preparation of the ‘live bio-therapeutic’ formulation and short-term viability testing of the product: **(A)** Flow chart of the ‘live bio-therapeutic’ preparation including reconstitution of freeze-dried or non-freeze dried *L. lactis* DPC3147 cells in either WFI or liquid paraffin/polysorbate 80. The formulation used in the animal trial is highlighted in red in the flow chart. Short-term viability of **(B)** non-freeze dried *L. lactis* DPC3147 cells stored in WFI; **(C)** non-freeze dried DPC3147 cells in an emulsion (‘live bio-therapeutic’ formulation used in this study); **(D)** freeze-dried DPC3147 cells stored in WFI and **(E)** freeze-dried DPC3147 cells in emulsion. Error bars represent standard deviations of mean values in each case.

A culture of *L. lactis* DPC3147 was maintained on LM17 agar (Merck KGaA, Darmstadt, Germany) at 30°C. A single colony of DPC3147 was inoculated into 10 ml LM17 broth, and incubated aerobically at 30°C under static conditions overnight. One milliliter of the overnight culture was sub-cultured into 100 ml LM17 broth, and incubated aerobically at 30°C overnight. This overnight culture was centrifuged at 8000 × *g* for 15 min at 4°C and the cell pellet was then washed twice with ice cold water for injection (WFI) (BioSciences Ltd., Dun Laoghaire, Dublin, Ireland), and concentrated ten-fold by suspending the cells in 10 ml WFI for downstream viability assays. A schematic of the preparation of the DPC3147 live bio-therapeutic formulation is included in Figure 1A.

### Preparation of Liquid Paraffin-Based Emulsion Containing *L. lactis* DPC3147 Cells (‘Live Bio-Therapeutic’)

Pharmaceutical grade liquid paraffin and polysorbate 80 were both purchased from Sigma Aldrich (Vale Road, Arklow, Ireland). Polysorbate 80 (10%) was prepared in WFI and sterilized by filtering twice through 0.2 μm syringe filters. Forty five ml of liquid paraffin was dispensed into 250 ml glass beakers and sterilized under dry heat at 170°C for 2 h and allowed to cool. Prior to preparation of the emulsion, the blade of an Ultra-Turrax^®^ homogenizer was steeped in 1% v/v Chlorus and UV-sterilized under laminar airflow for 1 h.

Ten ml cultures of *L. lactis* DPC3147 were grown overnight for 16 h at 30°C in LM17 broth. The cell pellet was isolated from overnight cultures by centrifuging at 8000 × *g* for 15 min at 4°C using a Thermo Scientific Sorvall centrifuge. The pellets were washed twice with 10 ml of WFI between centrifuging steps. The cells were finally reconstituted in 15 ml of 10% polysorbate 80. Forty five ml of pure sterile liquid paraffin was blended with 15 ml of the 10% polysorbate 80 (mixed with cells) at 10000 rpm in an Ultra-Turrax^®^ homogenizer under ice for 5 min to give a final cell concentration of approximately 1 × 10^9^ cfu per 5 ml dose (equivalent to 2 × 10^8^ cfu/ml) of the emulsion. *L. lactis* DPC 3147 in 10% polysorbate 80 was poured slowly into the liquid paraffin during blending. Thus the emulsion contained a final concentration of 2.5% polysorbate 80. A flow chart of the preparation of the ‘live bio-therapeutic’ emulsion formulation is included in Figure 1A.

### Preparation of Freeze-Dried Culture of *L. lactis* DPC3147

The culture was prepared as described above. After the final washing step, the culture was concentrated by a factor of 10 and was freeze-dried in 1 ml aliquots in 3 ml freeze-drying vials (Fisher Scientific, Ballycoolen, Dublin, Ireland) using a Virtis wiz 2.0 freeze dryer overnight. Freeze-dried cultures were reconstituted in either WFI or incorporated in the liquid paraffin emulsion (with a final concentration of 2.5% polysorbate 80). Pure sterile liquid paraffin was blended with freeze dried *L. lactis* cells re-suspended in a final concentration of 2.5% pharmaceutical grade polysorbate 80 at 10000 rpm in an Ultra-Turrax^®^ homogenizer on ice for 5 min. *L. lactis* in 10% polysorbate 80 was poured slowly into the paraffin during blending, as described above.

### *L. lactis* DPC3147 Cell Viability Testing

The viability of the freeze dried and non-freeze dried *L. lactis* DPC3147 cells in WFI and in the emulsion was examined after storage at 4°C, 22°C and 37°C. For short-term viability testing, plate counts from the samples were conducted daily for 5 days, while for long-term viability assays, plate counts were conducted every 7 days for up to 35 days. Following storage, the samples were serially diluted in maximum recovery diluent (MRD, Oxoid, Basingstoke, Hampshire, United Kingdom) and spread-plated on LM17 agar. Plates were incubated at 30°C for 24h and results expressed as cfu/ml.

### Cows Selected for “Live Bio-Therapeutic” Emulsion Formulation Trial, Intra-Mammary Infusion and Sampling

Holstein-Friesian dairy cows were first identified in the herd and animals were selected for the trial based on a SCC > 250,000 cells/ml. Cows with clinical mastitis exhibited obvious signs of inflammation and/or general malaise, clotted/abnormal milk production and the presence of pathogens in milk. Cows with sub-clinical mastitis did not display obvious signs of inflammation but had SCC > 250,000 cells/ml, clotted/abnormal milk and the presence of pathogens in milk. G^∗^Power calculations prior to conducting the trial revealed that a maximum of *n* = 20 cows per treatment group were sufficient for statistically valid results. To assess the efficacy of the live bio-therapeutic, cows that were presenting with high SCC were identified in the herd and the affected quarter infused with a one-time dose of the ‘live bio-therapeutic’ which was freshly made (approximately 1 × 10^9^ cfu of *L. lactis* DPC3147 per 5 ml dose; equivalent to 2 × 10^8^ cfu/ml) (*n* = 19 quarters from 18 cows). The control group received two doses of a commercial antibiotic (*n* = 18 quarters from 15 cows), which is used for routine treatment of lactating mastitic cows in the Moorepark herd at the Teagasc Animal and Grassland Research and Innovation Centre, Moorepark, Ireland, in accordance with good veterinary practice. This commercial antibiotic is a dual antibiotic product containing kanamycin (100,000 I.U.) and cephalexin (200 mg) and is active against *S. aureus, S. dysgalactiae, S. uberis* and *E. coli*. While previous field trials had utilized a commercial antibiotic formulation containing amoxycillin, clavulanic acid and prednisolone (Klostermann et al., 2008), here in this trial, we utilized Terrexine^TM^ for our control group, as this is the antibiotic formulation that is currently used to treat mastitis in the Moorepark herd.

All the treatments were administered directly into the teat sinus as described previously with minor modifications (Crispie et al., 2008; Klostermann et al., 2008). Briefly, prior to sampling, the teat was swabbed with 70% v/v ethanol on cotton wool. Immediately prior to infusion, a milk sample from the affected quarter was taken. Following this, 5 ml of the emulsion-based ‘live bio-therapeutic’ formulation containing approximately 1 × 10^9^ cfu per 5 ml dose (equivalent to 2 × 10^8^ cfu/ml) was infused into the mammary gland using sterile blunt-ended steel syringes (17 mm in length). One pre-loaded syringe of the commercial antibiotic was infused at the morning and evening milkings as per manufacturer’s instructions on the day of treatment. Milk samples were taken after 6h and then at intervals up to 7 days post-infusion for analysis of SCC, interleukin (IL)-8 titres, presence of mastitis pathogens and viable counts of *L. lactis* DPC3147 in milk.

### Somatic Cell Count (SCC) Measurements

SCC of milk samples were quantified as described previously with a few minor modifications (Klostermann et al., 2008, 2010). Briefly, milk samples were taken from the relevant quarters at time 0 (just before treatment) and at subsequent time points post-treatment. SCC determined in raw milk were measured using a Somacount 300^®^(Bentley Instruments Incorporated, United States).

### Quantification of IL-8 Levels in Milk Samples Using ELISA

Milk samples were taken as described above and centrifuged at 44000 × *g* for 30 min to separate the fat layer from the milk, which was removed using a sterile spatula. After centrifuging, the supernatants from the milk samples were subjected to IL-8 analysis by enzyme-linked immunosorbent assay (ELISA). The bovine IL-8 (CXCL8) ELISA development kit (Mabtech AB, Nacka Strand, Sweden) was used to measure IL-8 levels in milk samples taken at different time points pre- and post-treatment from cows treated with the ‘live bio-therapeutic’ emulsion preparation or the commercial antibiotic control. The following procedure was used: A 96-well Nunc-Immuno plate (Thermo Scientific, Ballycoolen, Dublin, Ireland) was coated with 100 μl of mAb MT8H6 at a concentration of 2 μg/ml (diluted in PBS pH 7.4) and the plate was kept overnight in the dark for 16h at 4°C. The following day, all wells were washed twice with 200 μl phosphate buffered saline (PBS). The plate was subsequently blocked with 200 μl PBS (supplemented with 0.05% Tween 20 and 0.1% bovine serum albumin) and the plate incubated at room temperature in the dark for 1 h. After 1h, the plate was washed five times using PBS supplemented with 0.05% Tween 20 (wash buffer). Following this, 100 μl of IL-8 standards ranging from concentrations of 12.5 pg/ml to 800 pg/ml were added to the wells in triplicate. Milk samples taken at different time points were diluted up to 100-fold in PBS and 100 μl of the undiluted samples, 1:10 dilutions and 1:100 dilutions were added to the wells in the microtiter plate in triplicate, and the plate incubated at room temperature in the dark for 2h. After 2h, each well was washed five times with wash buffer. Following these wash steps, 100 μl of mAb 26E5-biotin at a final concentration of 0.1 μg/ml was added to each of the wells and the plate incubated at room temperature in the dark for 1h. As before, the wells were washed with wash buffer five times. One hundred microliters of Streptavidin-ALP diluted 1:1000 was added to each of the wells and the plate incubated at room temperature in the dark for 1h, followed by washing five times. Finally, phosphatase substrate (Sigma Aldrich, Vale Road, Arklow, Ireland) at a final concentration of 1 mg/ml was prepared in de-ionized water supplemented with 7.5 mg/ml glycine, 1 mM ZnCl_2_ and 1 mM MgCl_2_ and the pH adjusted to pH 10.4. One hundred microliters of this substrate solution was added to each well and the plate incubated at 37°C in the dark for 1h. Absorbance readings at 405nm were taken using a plate reader (Synergy HT, BioTek). IL-8 values were interpolated using GraphPad Prism software (version 7.0) with 5-parameter logistics.

### *L. lactis* DPC3147 Clearance From Milk and Detection of Pathogens in Milk

Milk samples were serially diluted in MRD and 50 μl spread onto the surface of LM17 agar plates and incubated at 30°C for 24 h. The cultures were then overlaid with LM17 (1% agar) containing 1% v/v culture of the lacticin 3147-sensitive indicator strain *L. lactis* HP and incubated overnight at 30°C. Small white colonies producing a zone of inhibition against the *L. lactis* HP overlay were identified as *L. lactis* DPC3147 and cfu/ml of DPC3147 remaining in the milk were determined. The mean cfu/ml values and standard error of mean (SEM) values were determined. Plating of pathogens from raw milk samples obtained from high SCC cows was done using Blood agar (Oxoid Ltd., Basingstoke) (Table 1).

**TABLE 1:** Cure rates for cases which gave culture-positive milk samples.

	**Culture-positive**	**Identity of pathogens**	**Cure rate of culture-positive cases based on**
**Treatment**	**cases**		**SCC results (%)**
“Live bio-therapeutic” emulsion (total *N* = 19 quarters)	12/19 (63.16%)	*S. aureus* (*N* = 11), *Strep. uberis* (*N* = 1)	*S. aureus* 5/11 (45.45%), *Strep. uberis* 1/1 (100%), Culture-negative cases 3/7 (42.86%)
Antibiotic (total *N* = 18 quarters)	12/18 (66.66%)	*S. aureus* (*N* = 8), *Strep. uberis* (*N* = 2), *Strep. dysgalactiae* (*N* = 1), *E. coli* (*N* = 1)	*S. aureus* 4/8 (50%), *Strep. uberis* 0/2 (0%), *Strep. dysgalactiae* 0/1 (0%), *E. coli* 0/1 (0%), Culture-negative cases 5/6 (83.33%)

*The culture of pathogens from cows with mastitis was not successful in every case. *Staphylococcus aureus* was assumed to be the most probable cause of the high SCC for milk samples that were found to be culture-negative, on the basis of data collated from routine screening of milk samples from cows with clinical and sub-clinical mastitis in the Moorepark herd in Teagasc Animal and Grassland Research and Innovation Centre over the years. N = number of quarters infused. Percentages are indicated in parentheses.*

### Statistical Analysis

All of the statistical analyses were performed in Graph Pad Prism v7.0. Comparison of the cure rate of the two treatment groups was performed using a two-tailed Chi square test. Comparison of the ‘live bio-therapeutic’ formulation at different temperatures was done using Two-way ANOVA with Bonferroni corrections. A one-tailed Mann Whitney test was used to assess if a significant difference existed between the ‘live bio-therapeutic’ formulation and antibiotic treatment in the secretion of IL-8 and SCC/ml in the milk over the course of the experiment.

## Results

The overall aim of this study was to develop a ‘live bio-therapeutic’ formulation that could be practically administered on the farm and to compare this to a commercial antibiotic formulation routinely used to treat mastitis (Terrexine^TM^). In order to achieve this, we developed a liquid paraffin-based tube formulation, similar to tube formulations commonly used for antibiotic administration.

### Short-Term Testing of *L. lactis* DPC3147 in Various Formulations Reveals Cells Are Viable for at Least 5 Days

Viability of the *L. lactis* DPC 3147 cells was investigated daily for up to 5 days in four formats: (i) non-freeze dried cells re-suspended after centrifugation in WFI (Figure 1B); (ii) non-freeze dried cells re-suspended in a liquid paraffin/polysorbate 80-based emulsion (Figure 1C, ‘live bio-therapeutic’ formulation); (iii) freeze-dried cells re-suspended in WFI (Figure 1D) and iv) freeze-dried cells in a liquid paraffin/polysorbate 80-based emulsion (Figure 1E). Previous pilot trials had shown that approximately 10^9^ cfu per 5 ml dose was sufficient to elicit neutrophil influx into the mammary gland (Crispie et al., 2008). On that basis, we selected 1 × 10^9^ cfu/5 ml dose (equivalent to 2 × 10^8^ cfu/ml) as we hypothesized that such a concentration would evoke a sufficiently potent immune response, without causing any unexpected adverse reactions in the cows. The results from the short-term viability assays show that at 4°C, all the cell preparations maintained viable cells for up to 5 days (Figures 1B–E). Non-freeze dried cells in the emulsion (Figure 1C) displayed better viability than corresponding non-freeze dried cells in WFI (Figure 1B) when stored at 22°C and 37°C. The viability of freeze-dried cells in emulsions stored at 22°C and 37°C (Figure 1E) were more stable than corresponding freeze-dried cells stored in WFI (Figure 1D). Both non-freeze dried (Figure 1B) and freeze-dried cells stored in WFI (Figure 1D) exhibited a drop in viable cells counts within 5 days of storage at 22°C and 37°C.

### Liquid Paraffin Emulsion-Based Formulation of DPC3147 Maintains Viable Cells for Up to 5 Weeks

**FIGURE 2:**
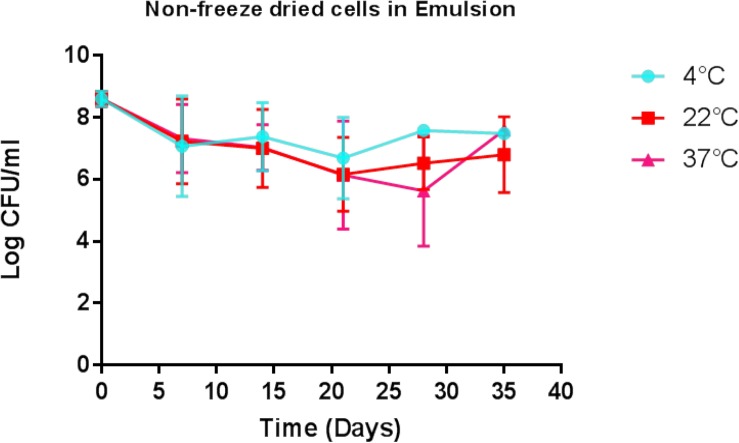
Viability of the ‘live bio-therapeutic’ formulation in a liquid paraffin/polysorbate 80-based emulsion prepared from washed cell preparation and stored at 4, 22, and 37°C. Determination of viability of *L. lactis* DPC3147 cells in a liquid paraffin-based emulsion (with a final concentration of 2.5% polysorbate 80) (“live bio-therapeutic’ formulation). Error bars represent standard deviations of mean values in each case.

Determination of the viability of the liquid paraffin-based emulsion containing *L. lactis* DPC3147 (‘live bio-therapeutic’ formulation), was assessed by conducting viable plate counts every 7 days, and showed that the cells remained viable to a high level for up to 5 weeks. With a starting concentration of approximately 10^9^ cfu/5 ml dose (2 × 10^8^ cfu/ml), cells remained at approximately 10^7^ cfu/ml even after 5 weeks, when stored at 4°C (Figure 2). Cell numbers fell to approximately 10^6^ cfu/ml at 5 weeks, when stored at 22°C or 37°C (Figure 2). In this study, approximately 1 × 10^9^ cfu/5 ml dose was selected to ensure a sufficiently potent immune reaction, based on previous pilot trials. Two-way ANOVA with Bonferroni corrections showed that there was no statistically significant difference between the mean log cfu/ml values for emulsion preparations stored at 4°C versus 22°C at any of the time points. Similarly there was no difference between the means of log cfu/ml values of samples stored at 4°C versus 37°C at any time point (*P* > 0.05 in all cases) with the exception of day 28 (*P* < 0.05).

### The “Live Bio-Therapeutic” Formulation Displays Comparable Efficacy to a Commercial Antibiotic Product in Curing Mastitis

**FIGURE 3:**
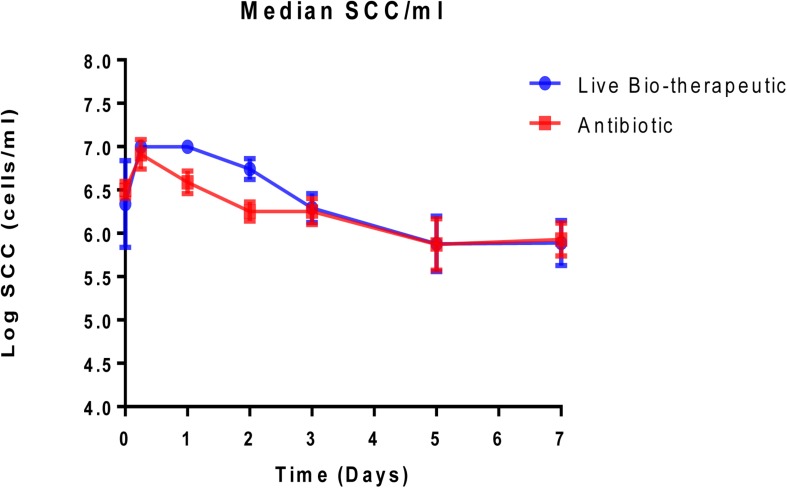
Median log somatic cell counts/ml in milk samples from groups of cows with mastitis treated with either the “live bio-therapeutic” formulation or the commercial antibiotic. Determination of SCC from milk samples obtained from cows treated with either the “live bio-therapeutic” emulsion or the commercial antibiotic. Error bars represent standard error for each of the treatment groups.

Animals with a SCC of >250,000 cells/ml (median SCC of 2,845,929 cells/ml) were selected for this study as they were representative of clinical or sub-clinical mastitis. These affected teats were infused with either *L. lactis* DPC3147 in a liquid paraffin-based emulsion (‘live bio-therapeutic’ formulation) or the control commercial antibiotic. While a minimum SCC of >250,000 cells/ml was chosen as a criterion for selecting cows with clinical/sub-clinical mastitis, the vast majority of animals selected had starting SCC significantly higher than this minimum value (as reflected by a relatively high median SCC value of 2,845,929 cells/ml). Cows were sampled at various time points over the course of 7 days and cure was considered to be a reduction in SCC from initially high starting counts (since median SCC of 2,845,929 cells/ml were approximately 10-fold higher than final counts after treatment) to an SCC of between 250,000 and 350,000 cells/ml within 5–7 days post-treatment. It was shown that the efficacy of the ‘live bio-therapeutic’ formulation is similar to the antibiotic (47% vs. 50% cure rate respectively, *P* = 0.93, Chi square test) (Figure 3 and Table 2). Initially after treatment, the ‘live bio-therapeutic’ group had a significantly higher median SCC than the antibiotic treatment group (*P* < 0.05, one-tailed Mann Whitney test, GraphPad Prism version 7.0) presumably due to the influx of neutrophils into the mammary gland. However, following 2 days post-infusion, the difference in the median of the SCC of both treatment groups was not significant (*P* > 0.05).

**TABLE 2:** Cure rates for the commercial antibiotic control and the ‘live bio-therapeutic’ preparation in emulsion.

**Treatment**	***N* (number of**	**Cure rate (%)**
	**quarters)**	
Commercial antibiotic	18	50
“Live bio-therapeutic” emulsion preparation	19	47

*Cure was considered to be a reduction of SCC to between 250,000 and 350,000 cells/ml within 5–7 days post-treatment.*

### The “Live Bio-Therapeutic” Formulation Evokes a Relatively Stronger Immune Response Compared to a Control Commercial Antibiotic Product

**FIGURE 4:**
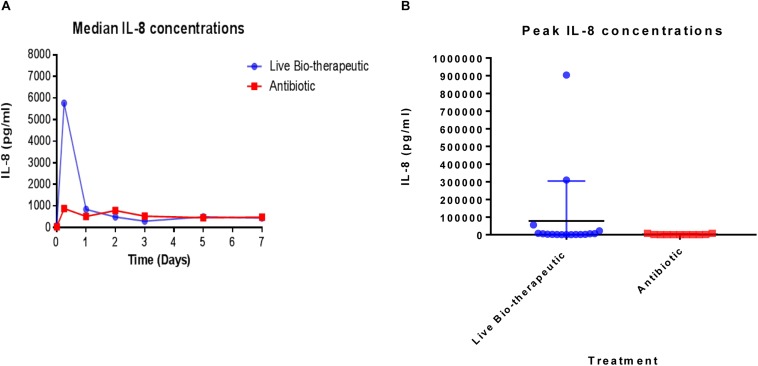
IL-8 concentrations quantified in milk samples from mastitic cows treated with the ‘live bio-therapeutic’ formulation or the commercial antibiotic. **(A)** Determination of median IL-8 titres (pg/ml) by ELISA from various milk samples obtained from different cows over time. These cows displayed mastitis and were treated with either the ‘live bio-therapeutic’ emulsion or the commercial antibiotic. **(B)** Peak IL-8 concentrations in milk samples obtained from various cows treated for clinical/sub-clinical mastitis with either the ‘live bio-therapeutic’ formulation or the commercial antibiotic. Each dot represents milk samples obtained from different quarters treated with the above-mentioned therapies.

In order to measure the immune response from the treatments, IL-8 levels were measured in the raw milk samples which were taken from various cows at different time points pre-and post-treatment (Figure 4). We selected IL-8 as a bio-marker since quantitative real-time PCR in previous trials had shown that IL-8 was the most up-regulated cytokine in response to the *L. lactis* live bio-therapeutic and other treatment groups. While there was a large inter-animal variation in IL-8 concentrations, overall, the peak concentrations, as well as the median values of IL-8 were greater in the group of animals treated with the ‘live bio-therapeutic’ preparation, indicating an increased localized immune response. The median IL-8 values of the ‘live bio-therapeutic’ group was significantly higher than the control antibiotic group (*P* = 0.0454, one-tailed Mann Whitney test, GraphPad Prism version 7.0, Figure 4A). Similarly, the median peak values of IL-8 for the ‘live bio-therapeutic’ group were also significantly higher than the median peak IL-8 values for the control antibiotics group (*P* = 0.0042, one-tailed Mann Whitney test, GraphPad Prism version 7.0, Figure 4B). In general, the range of IL-8 concentrations were highest within the first 72 h for both treatment groups following infusion and subsequently reduced with time, indicating that the most profound immune response was elicited during this time period for both treatment groups. These results strongly suggest that the mechanism of action of the ‘live bio-therapeutic’ formulation involves a stimulation of the immune system as indicated by the heightened IL-8 response (Figure 4). This host reaction most likely eliminates the offending pathogen, so that the infection is reduced and the SCC level subsequently decreases to between 250,000 and 350,000 cells/ml within 5–7 days of treatment.

### *L. lactis* DPC3147 Is Undetectable in Milk After 5 Days

**FIGURE 5:**
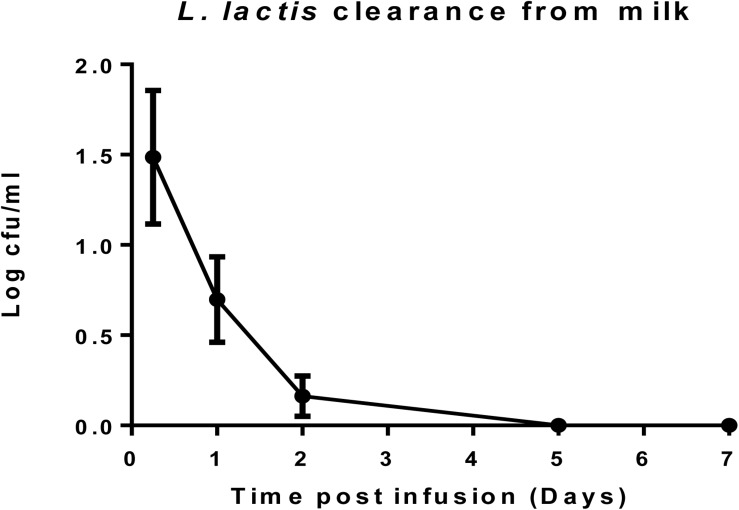
*L. lactis* DPC3147 clearance from milk. Determination of the rate of clearance of *L. lactis* DPC3147 from milk samples from a selection of cows treated with the ‘live bio-therapeutic’ formulation in emulsion. Graph shows mean values and standard error of mean (SEM).

After cows were infused with the ‘live bio-therapeutic’ preparation, the raw milk samples were tested at intervals for the presence of viable cells of *L. lactis* DPC3147. The data showed that no culturable cells of DPC3147 were detected in the milk five days post-infusion and that there were on average <10 cfu/ml in milk just after 1 day of treatment. These results demonstrate that the animals shed the ‘live bio-therapeutic’ in the milk in a very short time frame. It is likely that the host immune response, which is particularly potent within 6–24 h post-infusion, also results in the clearance of the DPC3147 cells from the teat, resulting in the relatively low numbers excreted in the milk. Since the infusion is with initially metabolically active DPC3147 cells, it is likely that the cells initially begin to produce lacticin 3147 in these conditions within the teat. We hypothesize that the concentrations of lacticin 3147 built up within the teat remain relatively low. However, the metabolically active cells initially infused likely induce a potent immune response involving IL-8, which helps to resolve the infection by clearing the offending pathogen. Overall, the relatively quick clearance rate, compounded by the fact that *L. lactis* has been granted GRAS status as a dairy starter organism, indicates that milk would not have to withheld post-treatment, unlike antibiotic treatment, which often results in milk withholding delays (Figure 5). Although the detection of viable but non-culturable (VBNC) populations of DPC3147 excreted in milk was beyond the scope of this study, we hope to investigate such intermediate viable sub-populations in future trials by using complementary techniques such as flow cytometry, in addition to viable plate counts.

### The Live Bio-Therapeutic Formulation Demonstrates Similar Efficacy to a Commercial Antibiotic Product Against Specific Pathogens

*Staphylococcus aureus* was found to be the predominant etiological agent for mastitis in both the live bio-therapeutic as well as the Terrexine^TM^ treatment groups. Overall, it was shown that the efficacy of the live-biotherapeutic in cows infected with *S. aureus* (45.45% cure rate) was comparable to the Terrexine^TM^ treatment group (50% cure rate). Further follow-up trials with a greater N number will provide insights into the relative efficacy of the live bio-therapeutic and Terrexine^TM^ against specific mastitis-causing pathogens.

## Discussion

In this study, we evaluated the effectiveness of a ‘live bio-therapeutic’ (an emulsion-based formulation of non-freeze dried *L. lactis* DPC3147) compared to a commercial antibiotic control for treating cows with clinical/sub-clinical mastitis. We selected non-freeze dried cells for the ‘live bio-therapeutic’ emulsion formulation primarily due to the relative ease of formulating freshly prepared emulsions containing non-freeze dried cells, compared to freeze-dried cells. While previous studies by our group had already highlighted the efficacy of DPC3147 aqueous-based formulations as potential therapeutic options for curing mastitis, some key objectives of the present study were (i) to develop and evaluate a formulation that would be ready to use by the farmer; (ii) to compare a single application of the ‘live bio-therapeutic’ formulation to a double application of a commercial antibiotic; (iii) evaluate the efficacy of the ‘live bio-therapeutic’ formulation against a commercial antibiotic which is commonly used on Irish farms and finally iv) to follow the fate of the introduced live bio-therapeutic following application.

The long-term viability of such a live bio-therapeutic emulsion is likely to be a crucial factor governing the success of the live bio-therapeutic as an alternative therapeutic agent. In this regard, it must be noted that strategies involving liquid paraffin-based emulsions have already been exploited for encapsulating other products including antibiotics, non-steroidal anti-inflammatory drugs (NSAIDS), cisplatin and insulin (Giri et al., 2013). In order for our emulsion-based ‘live bio-therapeutic’ formulation to be successful, the *L. lactis* cells must diffuse out of the droplets quickly into the infected mammary gland to evoke a potent immune response. An important factor determining diffusion rate in an emulsion is the volume of the droplets, due to the higher volume: surface area ratio of the droplets. It was encouraging to note in this present study that the viability of *L. lactis* DPC3147 remained relatively high in the ‘live bio-therapeutic’ formulation, for a period of 5 weeks when stored at 4, 22, and 37°C, indicating that the cells remain viable embedded in the lipid droplets. A long-term viable product of this nature could prove to be highly beneficial to farmers attempting to cure mastitis and is under development in our laboratory. It should also be emphasized that this product when applied in applicator tubes is in a ‘ready to use’ form by the farmer and can be introduced into the animal without any modification. However, as the properties of the bacterial cell surface might be altered following a period of prolonged storage, it is envisaged that the focus of future field trials in our group will involve testing the efficacy of such emulsion-based formulations after long-term storage, compared to freshly prepared emulsions of the same strain.

As a means of evaluating the success of the ‘live bio-therapeutic’ formulation, we assessed the effects of this emulsion-based formulation and the commercial antibiotic control on the SCC of cows presenting with clinical/sub-clinical mastitis in this trial. SCC is the standard bio-marker associated with mastitis infection and can be used as an indication of disease (Klostermann et al., 2008). Our group had previously demonstrated that intra-mammary infusion with viable *L. lactis* DPC3147 in WFI was effective at treating sub-clinical and clinical mastitis, with up to 60% cure rates in terms of eliminating signs of clinical mastitis, which was comparable to antibiotic efficacy in the trial (Klostermann et al., 2008). On this basis, we evaluated whether our novel emulsion-based formulation of viable DPC3147 cells was also effective in treating cows with mastitis. Similar to the findings of Klostermann and co-workers, we found that the cure rate of cows treated with the ‘live bio-therapeutic’ (47% cure rate) was comparable to commercial antibiotics (we obtained a 50% cure rate for the commercial antibiotic control group in this study, which was the expected cure rate), in terms of reducing SCC/ml five to 7 days post-infusion. In the present study, we noted an initial spike in SCC/ml 6-24h after infusion of the ‘live bio-therapeutic’ formulation, which indicates an induction of a localized immune response. The SCC counts returned to pre-infusion levels or lower than initial levels, within 7 days of treatment, which corroborated previous findings describing transient elevations in SCC counts shortly after infusion (Crispie et al., 2008; Klostermann et al., 2008; Beecher et al., 2009).

With respect to the mechanism of action of *L. lactis* DPC3147 cells in potentially treating mastitis, a previous study by Crispie et al., showed that unlike DPC3147 cells, intra-mammary infusion of cell-free supernatant (CFS) from lacticin 3147-producing cultures did not evoke a potent immune response (Crispie et al., 2008). Therefore, we hypothesized that the immune-stimulatory effect is likely to be predominantly cell-associated and not solely due to the presence of the bacteriocin. With this in mind, we compared the IL-8 titres in milk samples from cows treated with the ‘live bio-therapeutic’ emulsion containing viable *L. lactis* DPC3147 cells to the control commercial antibiotic in this trial. IL-8 was chosen as it was the most up-regulated cytokine in milk upon infusion with the ‘live bio-therapeutic’ in previous trials (Crispie et al., 2008). Despite a large inter-animal variation in IL-8 titres in milk as a result of ‘live bio-therapeutic’ infusion, the results demonstrate that the peak concentrations as well as the median values of IL-8 were greater in cows treated with this ‘live bio-therapeutic’ preparation compared to the commercial antibiotic group, suggesting a more potent immune response elicited by DPC3147 cells. In general, the range of IL-8 concentrations was highest within the first 72h after infusion and subsequently reduced with time, suggesting that the most profound immune response was evoked during this time period. This corroborated previous findings by Crispie et al., who demonstrated that intra-mammary infusion of DPC3147 induced the recruitment of neutrophils, providing an additional immunological defence against mastitis-causing pathogens. Indeed, a key function of IL-8 is to recruit polymorphonuclear leukocytes (neutrophils) as well as other immune cells to the site of infection, assisting in the resolution of the infection.

It is interesting to note that previous studies by Bannerman and co-workers, as well as Yang and co-workers, had demonstrated that while the Gram negative mastitis-causing pathogen *E. coli* elicited increases in IL-8 expression, perhaps the most common mastitis-causing pathogen, *S. aureus*, failed to trigger such an effect (Bannerman et al., 2004; Yang et al., 2008). Indeed, a failure to evoke a potent immune reaction in response to *S. aureus* may contribute to its widespread persistence as an etiological agent of recurrent mastitis. A study of a similar nature by Beecher and co-workers showed that while the pathogen *Strep. dysgalactiae* induced a modest increase in IL-8 gene expression, the ‘live bio-therapeutic’ *L. lactis* DPC3147 triggered a more potent immune response, compared to the pathogens tested in the study (Beecher et al., 2009). Thus, the immune-stimulatory activity of ‘live bio-therapeutic’ containing viable *L. lactis* DPC3147 cells is likely to be the main mechanism of action which explains its efficacy in this and previous trials. Although the concentration of lacticin 3147 produced by the DPC3147 cells in the teat was not measured in this trial, we hypothesize that the concentrations would be relatively low upon infusion, as the DPC3147 cells would require enough time to produce sufficient concentrations of the peptides to kill the offending pathogen. The concentrations of bacteriocin produced in the teat will have to be quantified in future field trials of this nature, perhaps by measuring the concentrations excreted in milk, in order to gain insights as to whether the presence of the bacteriocin peptides contribute to the immune-stimulatory effect and apparent resolution of the disease or not. In addition, follow-up field trials are currently being conducted, testing the effects of a bacteriocin-negative derivative of *L. lactis* DPC3147 (i.e., *L. lactis* DPC 5399, devoid of the pMRC01 plasmid and the lacticin 3147 biosynthetic machinery) to elucidate whether bacteriocin production is vital for inducing an immune response or not. Irrespective of bacteriocin production, we hypothesize that the concentrations of the peptides present in the teat are relatively low and at sub-inhibitory concentrations, and thus unlikely to exert a direct antimicrobial effect on the offending pathogen. This warrants further investigation in future field trials, which will focus on precisely quantifying the concentrations of the peptides *in vivo* to fully unravel the complex mechanism of action of treating this disease.

Overall, the potential use of the ‘live bio-therapeutic’ as a viable alternative *in lieu* of commercial antibiotics presents some notable advantages. Significantly, several commercial antibiotic formulations must be administered using two doses, which frequently results in delays in the milking process to re-commence. In contrast, by utilizing a single dose of the ‘live bio-therapeutic’ formulation, one could hasten the re-commencement of milking, in turn resulting in reduced economic losses. In addition, the current practice of discarding milk following antibiotic treatment due to the presence of residues is unlikely be an issue following treatment with the ‘live bio-therapeutic’ formulation since the organism is food-grade. Moreover, in this trial, we have demonstrated that DPC3147 cells are completely cleared from milk within 5 days of infusion, most likely as a consequence of the immune response. Furthermore, the relatively inexpensive nature of the ‘live bio-therapeutic’ formulation can help to reduce overall costs involved in treating bovine mastitis. Despite its history of safe use as a dairy starter organism, the strain *L. lactis* DPC3147 must fulfill a number of criteria before being granted Qualified Presumption of Safety (QPS) status by the European Food Safety Authority (EFSA) as a bio-therapeutic. One key example of such a criterion includes the absence of transmissible antibiotic resistance genes on mobilizable elements. In addition, the strain must not possess any virulence factors or have the propensity to cause any opportunistic infections, whilst being considered for QPS status as a bio-therapeutic.

In conclusion, we have developed a ready to use formulation which proved as efficacious as antibiotic treatment for mastitis treatment. The findings described in this study warrant further investigations in future field trials to fully unravel the complex mechanisms of action involved in the treatment of bovine mastitis. Indeed, an assessment of the efficacy of a heat-killed emulsion-based formulation of the DPC3147 is the focus of a follow-up field trial in our group. We hope that this will provide insights into the mechanism of action of the product and whether the cells have to be viable or not to elicit an equally potent immune-stimulatory effect. An equally potent immune-stimulatory effect induced by heat-killed cells of the strain would obviate the need to prepare fresh viable cells and any concerns regarding the long-term viability of the cells. Furthermore, future field trials of this nature warrant precise identification of the offending pathogen using DNA-based methodology, as the identification of pathogens using a culture-based method is rather limited. The creation of bio-banks of mastitis-causing pathogens, and precise identification of such strains as part of future trials, would help in determining the lacticin 3147 sensitivity/resistance patterns of these pathogenic strains. Finally, subject to ethical approval by local Animal Ethics Committees, we hope to investigate whether administering two injections of the live bio-therapeutic formulation proves more efficacious than a single dose, as part of future trials. Nonetheless, overall, the implications of the findings described here could eventually lead to a significant reduction of antibiotics for treatment of this most persistent disease in dairy cows.

## Data Availability Statement

The datasets generated for this study are available on request to the corresponding author.

## Ethics Statement

This field trial was approved by the Teagasc Animal Ethics Committee (TAEC) and the Health Products Regulatory Authority (HPRA). G^∗^Power calculations were conducted to determine the optimum number of animals required for the trial, keeping in line with the principles of the 3Rs (replacement, reduction, and refinement).

## Author Contributions

MK and HM are researchers in APC Microbiome Ireland and Moorepark Food Research Centre, Teagasc, drafted the manuscript. MK optimized the ‘live bio-therapeutic emulsion’ formulation and conducted viability and stability testing and ELISAs. HM conducted long-term viability assays and ELISAs. JF conducted the SCC determinations and plating of pathogens. NB performed the infusions. JF, NB, PD, RS, MR, CH, and RR revised the manuscript. All authors read and approved the final manuscript.

## Conflict of Interest

The authors declare that the research was conducted in the absence of any commercial or financial relationships that could be construed as a potential conflict of interest.

## Abbreviations

AMR: antimicrobial resistance
APP: acute phase proteins
CFS: cell-free supernatant
cfu: colony forming unit
ELISA: enzyme-linked immunosorbent assay
GRAS: generally regarded as safe
IL-8: interleukin 8
MRD: maximum recovery diluent
NSAID: non-steroidal anti-inflammatory drugs
PBS: phosphate buffered saline
SCC: somatic cell counts
WFI: water for injection
WHO: World Health Organization
